# Dysmenorrhea and Occupational Factors: Korea Nurses' Health Study

**DOI:** 10.1155/jonm/1968522

**Published:** 2024-11-13

**Authors:** Heeja Jung, Hyunju Dan, Chiyoung Cha, Yanghee Pang

**Affiliations:** ^1^College of Nursing, Konyang University, Daejeon, Republic of Korea; ^2^Department of Nursing, Hwasung Medi-Science University, Hwasung-si, Republic of Korea; ^3^College of Nursing, Ewha Womans University, Seoul, Republic of Korea; ^4^Department of Nursing, Seoil University, Seoul, Republic of Korea

**Keywords:** dysmenorrhea, Korea Nurses' Health Study, nurses, occupational characteristics, women of childbearing age

## Abstract

**Aim:** To examine the prevalence of dysmenorrhea in the predominantly female nursing profession and investigate the role of occupational characteristics in dysmenorrhea.

**Background:** Studies on working women have mostly examined the effects of dysmenorrhea symptoms on work performance, as opposed to shedding light on the association between work-related characteristics and dysmenorrhea.

**Methods:** In this cross-sectional study, we used data obtained from survey 9 of the Korea Nurses' Health Study. The participants were female nurses of childbearing age. Statistical analysis included descriptive statistics and multivariable logistic regression.

**Results:** Data from 6697 participants were analyzed. Of the total sample, 47.3% had dysmenorrhea. After adjusting for confounders to examine the relationship between occupational characteristics and dysmenorrhea, the odds for dysmenorrhea were 1.230 times higher among women who lifted heavy objects at least six times a day compared to those who did not engage in heavy lifting (95% confidence interval: 1.028–1.473) and 1.042 times higher among women with higher physical fatigue (odds ratio: 1.042, 95% confidence interval: 1.023–1.061).

**Conclusion:** The findings clarify the potential for reducing dysmenorrhea through the improvement of work environment factors. Thus, this study may prove useful for developing educational programs and policies that aim to alleviate dysmenorrhea among working women, including nurses.

**Implications for Nursing Management:** Nursing managers and health policymakers need to understand the factors influencing dysmenorrhea and minimize female nurses' physical burden by implementing appropriate nurse–patient ratios and improving their work environment.

## 1. Introduction

Dysmenorrhea—lower abdominal pain that occurs before or during menstruation, accompanied by symptoms such as sweating, headaches, nausea, vomiting, diarrhea, and tremulousness—is the most common gynecological disorder among women of childbearing age [1]. Dysmenorrhea is divided into two major categories: primary and secondary. Primary dysmenorrhea is the presence of recurrent, crampy pelvic pain in the lower abdomen that occurs just before and/or during menstruation in the absence of suspected or proven pelvic pathology. Secondary dysmenorrhea is painful menstruation in the presence of a disease that causes the recurrent, cyclic pain symptoms, such as endometriosis, adenomyosis, or uterine leiomyomata [2]. The prevalence of primary dysmenorrhea varies according to literature; however, the studies since 2020 have shown the prevalence to be 78%–92.5% in the adolescent group [3, 4], 66.1%–91.5% in the female college student group [5, 6], and 79%–92.3% in the entire female group of childbearing age [7, 8]. Particularly, a greater prevalence has been reported in the young female group, and the prevalence of dysmenorrhea in meta-analytic study of female college students has increased to 71.5% in studies between 2015 and 2021 compared to approximately 58.8% in a previous study in 2010, requiring continuous monitoring of the prevalence [5]. Dysmenorrhea symptoms adversely impact women's academic endeavors, work, and quality of life [9–12], potentially leading to absenteeism and reduced working efficiency among working women of childbearing age [10].

The major predictors of primary dysmenorrhea suggested in previous studies include age [4], parity [13], age at menarche, heavy menstrual flow [3, 8], regularity of menstruation [5, 8], length of menstrual cycle [8], chronic pelvic pain [3], gynecologic diseases [14], family history [5], body mass index (BMI) [15], alcohol consumption, smoking, food intake [5, 16], shift work [17], physical activity [5], sleep, and stress [5, 16]. Although a recent study showed that work-related characteristics such as high work intensity, prolonged standing or frequent heavy lifting, and night work are also associated with menstrual disorders, including dysmenorrhea [18], studies shedding light on the diverse predictors of dysmenorrhea among working women of childbearing age are lacking.

The nursing profession, which predominantly consists of women, is characterized by shift work, overtime, prolonged standing, and heavy lifting, as well as high physical and mental stress [19–21]. The prevalence of dysmenorrhea was reported to be 69.9% among nurses in Turkey [22], compared to 43% and 61.1% among sales workers and call center workers, respectively, who are subject to high emotional labor [23]. Owing to the nature of the profession, nurses experience high levels of emotional labor and physically demanding workloads as they provide direct care to patients and their families.

According to previous studies, the psychological distress of working women was significantly related to the severity of dysmenorrhea [24]; hence, the emotional labor of nurses can be considered a major influencing factor of dysmenorrhea. Additionally, rotating shift work women showed a higher degree of dysmenorrhea than those with fixed day work [17], and shift work is consistently reported as a major influencing factor of dysmenorrhea [25]. Occupational physical demand increases the intensity and frequency of dysmenorrhea [26]. Prolonged standing hours and frequent heavy lifting among female workers were found to be significantly related to menstrual disorders [27]. Thus, it is important to identify the predictors of dysmenorrhea among nurses and implement measures to address them.

Research on the predictors of dysmenorrhea has focused on primary dysmenorrhea among adolescents and female college students [9, 15] or among the entire population of women of childbearing age [11, 28], with only a handful of studies conducted on a specific subset of women, such as working women. Furthermore, even studies on working women have mostly examined the effects of dysmenorrhea on work performance, as opposed to shedding light on the association between work-related characteristics and dysmenorrhea [10, 29]. Some studies have examined the association between shift work and dysmenorrhea [17], but research examining the association of various work-related characteristics and dysmenorrhea among working women of childbearing age is scarce. In this context, we aim to examine the prevalence of dysmenorrhea in the predominantly female nursing profession and investigate its associations with reproductive health, lifestyle, and occupational characteristics.

## 2. Methods

### 2.1. Design

In this cross-sectional study, we used data obtained from the Korea Nurses' Health Study (KNHS). The KNHS is a national prospective study conducted on nurses in South Korea, and data are collected using the protocol and questionnaire from the US Nurses' Health Study 3, with necessary modifications in consideration of South Korea's culture and hospital organizational features. The purpose of the KNHS is to identify the effects of lifestyle, environmental characteristics, and occupational characteristics on health among women of childbearing age [30].

### 2.2. Participants

A simple random sampling was used for the initial recruitment. Out of 157,569 eligible participants, 20,613 participated in survey 1, which was higher than the target sample size [30]. A total of 20,613 female nurses aged 20–45 years who worked in a hospital in South Korea participated in the first survey of the KNHS (baseline data collection). Since that time, yearly follow-up surveys have been conducted. For the follow-up surveys, announcements on data collection with survey link were sent to the 20,613 female nurses who participated in survey 1. For survey 9, data were collected from October 2020 to April 2021 (*n* = 10,656).

The inclusion criteria for the present study were participants of survey 9 who had worked throughout the past year and had menstrual cycles in the past three months and had response about age of menarche (*n* = 7337). The exclusion criteria were women who took hormone contraceptives (*n* = 93), pregnant women (*n* = 456), women who had given birth in the past six months, and breastfeeding women (*n* = 91).

### 2.3. Measurements

The general characteristics assessed included age, educational level, marital status, and annual income. Reproductive health–related characteristics included age of menarche, length of menstrual cycle, regularity of menstruation, parity, endometriosis, and uterine myoma diagnosis. Age of menarche was divided into ≤ 12 years, 13–14 years, and ≥ 15 years; the length of menstrual cycle was divided into < 25 days, 26–31 days, and ≥ 32 days. Regularity of menstruation was divided into regular and irregular, and parity was divided into 0, 1, and ≥ 2.

Lifestyle factors included alcohol consumption, smoking, and BMI. Alcohol consumption and smoking were categorized into yes and no, and BMI was calculated by dividing body weight by height squared and categorized into < 18.5, 18.5–< 23, and ≥ 23 kg/m^2^. Sleep disturbance was assessed using the Jenkins Sleep Evaluation Questionnaire, which measures sleep problems experienced in the past four weeks [31]. It consists of four items, each rated on a 6-point Likert scale. The total score ranges from 0 to 20, and a higher score indicates more sleep disturbances. Cronbach's α was 0.850 in this study.

Occupational characteristics included shift work, weekly work hours, standing work hours, and frequency of lifting heavy objects over 10 kg. Weekly work hours were categorized into ≤ 40 and ≥ 41, and standing work hours per day were divided into < 1/day, 1–4/day, and ≥ 5/day. The frequency of lifting heavy objects over 10 kg was divided into none, 1–5 times/day, and ≥ 6 times/day. Fatigue was measured using the Chalder Fatigue Scale [32]. This self-report questionnaire consists of 11 items measuring physical and mental fatigue. Items 1–7 pertain to physical fatigue, and items 8–11 to mental fatigue. Each item is rated on a 4-point Likert scale. The physical fatigue and mental fatigue scores range from 0 to 21 and 0 to 12, respectively, with higher scores indicating greater fatigue. In the original study, the Cronbach's alpha values were 0.845 for physical fatigue and 0.821 for mental fatigue. Cronbach's α was 0.864 for physical fatigue and 0.843 for mental fatigue in this study.

Dysmenorrhea was defined based on the participant's answer of “yes” to the question “Do you have menstrual pain?” and the statement that they were taking pain relievers to alleviate the menstrual pain [33].

### 2.4. Ethical Considerations

This study was reviewed and approved by the Institutional Review Board of Ewha Womans University, the principal investors' institution (#ewha-201904-0012-09, #ewha-201904-0012-12), which waived the requirement for obtaining informed consent. The potential participants were able to review the informed consent form, which included the purpose of the study, measures for maintaining confidentiality, and the right to withdraw from the study at any time via mobile phone or the Internet before the survey. Their agreement to participate in the study was indicated by clicking the “agree” button. The researchers' information and access to an anonymous bulletin board were provided to participants who had questions about the study.

### 2.5. Statistical Analysis

Data were analyzed using the SPSS Statistics software Version 26.0 (IBM Corp., Armonk, New York, United States of America). Descriptive statistics, namely, frequency, percentage, mean, and standard deviation, were used. The participants' general characteristics, reproductive health, lifestyle, and occupational characteristics were analyzed using a chi-square test. Among the lifestyle and occupational characteristics, sleep disturbance, physical fatigue, and psychological fatigue were analyzed using *t*-test. The correlates of dysmenorrhea were identified using multivariable logistic regression analysis. Logistic regression was used to control for confounding variables and to provide an odds ratio (OR) adjusted for multiple confounders [34]. In this study, the general characteristics were included in Model 1, reproductive health characteristics in Model 2, lifestyle characteristics in Model 3, and occupational characteristics in Model 4. After adjusting for confounders, the association between occupational characteristics and dysmenorrhea was investigated. Statistical significance was set at *p* < 0.05 for all analyses.

## 3. Results

### 3.1. Participant Characteristics

Data from a total of 6697 participants were analyzed. Among the sample, 47.3% had dysmenorrhea, and the participants' general characteristics are shown in Table 1. Most participants (59.6%) were aged 30–39 years, and 65.7% were married, divorced, separated, or widowed. The results of the chi-square tests showed that age, education, annual income, and marital status were significantly different between nurses with dysmenorrhea and those without (*p* < 0.001). Compared with nurses without dysmenorrhea, those with dysmenorrhea were mostly aged 20–29 years (11.8% vs. 6.1%) and unmarried (43.9% vs. 25.8%). In terms of reproductive health, 82.5% had regular menstrual cycles, and 62.8% had a cycle lasting 26–31 days. Endometriosis and uterine myoma were diagnosed in 4.3% and 12.1% of the participants, respectively. Compared with nurses without dysmenorrhea, those with dysmenorrhea were more likely under 12 years of menarche age (24.8% vs. 20.3%), have over 32 days of menstrual cycle (25.1% vs. 21%), have not given birth (59.5% vs. 35.3%), and have endometriosis (5.2% vs. 3.4%). In terms of lifestyle, 74% had alcohol exposure, and 57% had a normal BMI (18.5–< 23 kg/m^2^). In terms of occupational characteristics, 45.2% of the participants worked shifts, and 57.3% worked 41 h or more per week. Participants with dysmenorrhea were more likely taking alcohol (75.7% vs. 72.5), underweight (8.2% vs. 6.6%), shift workers (50% vs. 40.9%), working more than 41 h (60% vs. 54.8%), and lifting objects over 10 kg (16.3% vs. 12.3%). Participants with dysmenorrhea scored higher in sleep disturbance, physical fatigue, and psychological fatigue than those without dysmenorrhea (Table 2).

### 3.2. Correlates of Dysmenorrhea

Multivariable logistic regression analysis was performed to identify the correlates of dysmenorrhea. In the final model, the odds for dysmenorrhea were lower among those aged ≥ 40 (OR: 0.529, 95% confidence interval (CI): 0.423–0.662), those who had age of menarche at ≥ 15 (OR: 0.842, 95% CI: 0.726–0.977), those who had irregular menstrual cycles (OR: 0.752, 95% CI: 0.647–0.874), those who had one child (OR: 0.566, 95% CI: 0.472–0.678) or two children (OR: 0.408, 95% CI: 0.342–0.487) compared to none, and those who were obese (OR: 0.877, 95% CI: 0.785–0.980). The odds for dysmenorrhea were 1.721 times higher among women diagnosed with endometriosis (95% CI: 1.331–2.226), 1.130 times higher among women with alcohol exposure (95% CI: 1.006–1.270), and 1.037 times higher among women with more sleep disturbances (95% CI: 1.023–1.051). In terms of occupational characteristics, the odds for dysmenorrhea were 1.230 times higher among women who lifted heavy objects at least six times a day compared to those who did not lift heavy objects (95% CI: 1.028–1.473) and 1.042 times higher among women with higher physical fatigue (95% CI: 1.023–1.061) (Table 3).

## 4. Discussion

We aimed to identify the prevalence of dysmenorrhea and its correlates among female nurses of childbearing age in South Korea. Previous studies have also reported a high prevalence of dysmenorrhea among nurses. Approximately 69.9% of Turkish nurses experience dysmenorrhea, of whom 86% had moderate or severe type [22]. The prevalence of dysmenorrhea among the Nepalese nurses was 77.8% [35]. In this study, dysmenorrhea was defined as taking medications for menstrual pain, and 47.3% of the participants had dysmenorrhea. This rate is higher than 14.5% reported among Chinese female nurses using the same criteria for assessing dysmenorrhea [18]. This finding indicates a high prevalence of dysmenorrhea among South Korean nurses. In a study examining the changes in labor intensity among South Korean nurses, weekly working hours and long working days tended to decrease, while work density and emotional labor tended to increase [36]. The increase in physical and emotional workloads may have contributed to the high prevalence of dysmenorrhea. Dysmenorrhea symptoms negatively affected the job satisfaction, work performance, and service quality of nurses [22, 37]. Therefore, addressing the factors that contribute to the increased incidence of dysmenorrhea among female nurses is crucial.

In the present study, the occupational characteristics of female nurses of childbearing age were included in the final model, and their associations with dysmenorrhea were examined after adjusting for confounders. The results indicated that shift work was not significantly associated with dysmenorrhea, which is consistent with the findings of a Spanish study conducted in nurses and nurse assistants [38]. However, a meta-analysis of the association between shift work and dysmenorrhea among working women revealed that dysmenorrhea was more prevalent among shift workers [17]. In this study, the presence or absence of shift work was evaluated, but the duration or pattern of shift work was not investigated. Hence, further studies are needed to determine whether the duration and type of shift work contribute to dysmenorrhea among working women. Jiang et al. [18] defined menstrual disorders as having at least one of the following symptoms: dysmenorrhea, heavy menstrual bleeding, irregular menstruation, or abnormal menstrual duration. They reported that the prevalence of menstrual disorders increases with longer working and standing hours. However, no significant association was observed between dysmenorrhea and working or standing hours after controlling for confounders. Previous studies grouped various symptoms under the term “menstrual disorders”; however, our study specifically investigated the direct relationship between working characteristics and dysmenorrhea, resulting in different findings.

Among occupational characteristics, heavy lifting (lifting an object weighing ≥ 10 kg) was significantly associated with the occurrence of dysmenorrhea. Previous studies have also reported that heavy lifting increases the likelihood of experiencing irregular menstruation [39, 40] and menstrual disorders among female nurses [18]. Heavy lifting appeared to have an adverse impact on women's menstrual health, highlighting the importance of preventing heavy lifting by female nurses in the workplace. Furthermore, the risk of dysmenorrhea increased with increasing physical fatigue, indicating a positive association between the physical work burden and dysmenorrhea. These results show that, alongside emotional labor [23], excessive physical labor, particularly heavy lifting and physical fatigue, may elevate the risk of dysmenorrhea.

As reported in previous studies, increasing age, age of menarche, and parity [3, 4, 13] decreased the risk of dysmenorrhea. However, contrary to the findings of earlier studies, which indicated a higher risk of dysmenorrhea in women with irregular menstruation [5, 16], we observed a greater risk among women with regular menstruation. This discrepancy may be attributed to the age difference between our participants, who were mostly 30 years or older, and the population in the previous study, which consisted only of students aged < 25 years. Further research is required to examine the relationship between regular menstruation and dysmenorrhea in women of childbearing age. This study confirmed the diagnoses of endometriosis and uterine myoma, both associated with secondary dysmenorrhea [41]. A previous study reported a 7.7% prevalence of pathological (secondary) dysmenorrhea in the childbearing age group [42]. In our study, 4.3% of the participants were diagnosed with endometriosis and 12.1% with uterine myoma, indicating a higher prevalence. Additionally, endometriosis showed a significant association with dysmenorrhea.

Among the lifestyle factors, the risk of dysmenorrhea was lower in women with a BMI indicative of obesity. A linked was found between dysmenorrhea and BMI in adolescents, indicating a high risk of dysmenorrhea among adolescents who are underweight [5, 15]. Therefore, investigating the relationship between dysmenorrhea and BMI in the entire female population is crucial. In our study, alcohol consumption was identified as a risk factor for dysmenorrhea, despite previous studies suggesting that alcohol might reduce dysmenorrhea [16]. As this study did not determine the frequency and amount of alcohol consumption, further studies are needed to clarify the relationship between alcohol consumption patterns and dysmenorrhea in women of childbearing age. Additionally, the risk of dysmenorrhea increased with greater sleep disturbances, aligning with the findings of a previous study [5] that linked sleep disturbances to dysmenorrhea among working women. Shift work and high work intensity, common among nurses, can affect sleep disorders [36]; hence, implementing appropriate staffing and shift work patterns is necessary.

### 4.1. Limitations

Despite several strengths of this study, the following are its limitations. First, as this study used a cross-sectional design, it is not possible to make causal inferences. Second, dysmenorrhea was classified as either present or absent; therefore, the severity and difference between primary and secondary menstrual cramps were not represented in the findings. Third, this study was unable to incorporate all major influencing factors of dysmenorrhea observed in previous studies as confounding variables to maintain the follow-up rate of participants in a cohort study by managing the number of survey questions. Consequently, it is imperative to address these variables in future research. Lastly, as the web-based survey was voluntary, it is not representative of the entire population of South Korean nurses.

## 5. Conclusion

We examined the relationship of dysmenorrhea with reproductive health and lifestyle and occupational factors. Occupational characteristics, such as heavy lifting and physical fatigue, were significantly associated with dysmenorrhea after adjusting for potential confounders. However, we did not observe the significant correlation of dysmenorrhea with shift work, working hours, and standing hours demonstrated in previous studies. Therefore, future studies targeting various factors such as shift work patterns and physical and emotional workload are needed. As the findings present potential methods for reducing dysmenorrhea by improving work environment factors, this study may be useful for developing educational programs and policies that aim to alleviate dysmenorrhea among working women, including nurses.

### 5.1. Implications for Nursing Management

Nursing professionals, managers, and health policymakers need to understand the factors associated with dysmenorrhea and implement appropriate educational programs such as those related to lifestyle improvement and alleviating occupational risk factors. Dysmenorrhea symptoms adversely impact women's work and quality of life and may also lead to reduced working efficiency among women of childbearing age. The potential negative impact of dysmenorrhea on nurses' personal health may influence their performance, and hence, the quality of patient care. Policymakers and hospital managers need to apply appropriate interventions for managing sleep quality and physical fatigue. Furthermore, there is a need to develop a work environment wherein female nurses are not required to engage in excessive heavy lifting and where their physical burden is minimized via the implementation of appropriate nurse–patient ratios.

## Figures and Tables

**Table 1:** Dysmenorrhea according to general and reproductive health characteristics (*N* = 6697).

Variables	*n* (%)	Dysmenorrhea		*χ* ^2^	*p*
		Yes (*n* = 3165)	No (*n* = 3532)		
Age (years)				341.263	< 0.001
20–29	589 (8.8)	374 (11.8)	215 (6.1)		
30–39	3990 (59.6)	2130 (67.3)	1860 (52.6)		
≥ 40	2118 (31.6)	661 (20.9)	1457 (41.3)		
Education				52.184	< 0.001
Diploma	1083 (16.2)	506 (16.0)	577 (16.3)		
Bachelor's	4226 (63.1)	2118 (66.9)	2108 (59.7)		
Master's or higher	1388 (20.7)	541 (17.1)	847 (24.0)		
Marital status				241.550	< 0.001
Unmarried	2299 (34.3)	1388 (43.9)	911 (25.8)		
Married or other	4398 (65.7)	1777 (56.1)	2621 (74.2)		
Annual income (USD)				28.295	< 0.001
< 30,000	656 (9.8)	304 (9.6)	352 (10.0)		
30,000–39,999	1797 (26.8)	945 (29.9)	852 (24.1)		
≥ 40,000	4244 (63.4)	1916 (60.5)	2328 (65.9)		
Age of menarche (years)				26.673	< 0.001
≤ 12	1501 (22.4)	784 (24.8)	717 (20.3)		
13–14	3407 (50.9)	1608 (50.8)	1799 (50.9)		
≥ 15	1789 (26.7)	773 (24.4)	1016 (28.8)		
Length of menstrual cycle (days)				16.213	< 0.001
≤ 25	953 (14.2)	435 (13.7)	518 (14.6)		
26–31	4208 (62.8)	1935 (61.2)	2273 (64.4)		
≥ 32	1536 (22.9)	795 (25.1)	741 (21.0)		
Regularity of menstruation				2.368	0.124
Regular					
Irregular					
Parity				441.160	< 0.001
0	3130 (46.7)	1883 (59.5)	1247 (35.3)		
1	1352 (20.2)	587 (18.5)	765 (21.7)		
≥ 2	2215 (33.1)	695 (22.0)	1520 (43.0)		
Endometriosis				13.045	< 0.001
Yes	286 (4.3)	165 (5.2)	121 (3.4)		
No	6411 (95.7)	3000 (94.8)	3411 (96.6)		
Uterine myomas				0.063	0.802
Yes	809 (12.1)	379 (12.0)	430 (12.2)		
No	5888 (87.9)	2786 (88.0)	3102 (87.8)		

**Table 2:** Dysmenorrhea according to lifestyle and working characteristics (*N* = 6697).

Variables	*n* (%) or M ± SD	Dysmenorrhea		*χ* ^2^/*t*	*p*
		Yes (*n* = 3165)	No (*n* = 3532)		
Alcohol consumption				9.193	0.002
Yes	4957 (74.0)	2397 (75.7)	2560 (72.5)		
No	1740 (26.0)	768 (24.3)	972 (27.5)		
Smoking				3.261	0.071
Yes	180 (2.7)	97 (3.1)	83 (2.3)		
No	6517 (97.3)	3068 (96.9)	3449 (97.7)		
BMI (kg/m^2^)				22.999	< 0.001
< 18.5	492 (7.3)	260 (8.2)	232 (6.6)		
18.5–< 23	3814 (57.0)	1863 (58.9)	1951 (55.2)		
≥ 23	2391 (35.7)	1042 (32.9)	1349 (38.2)		
Shift work				56.238	< 0.001
Yes	3029 (45.2)	1584 (50.0)	1445 (40.9)		
No	3668 (54.8)	1581 (50.0)	2087 (59.1)		
Weekly work hours				18.351	< 0.001
≤ 40	2862 (42.7)	1266 (40.0)	1596 (45.2)		
≥ 41	3835 (57.3)	1899 (60.0)	1936 (54.8)		
Standing work (hours)				2.072	0.355
< 1	608 (19.2)	608 (19.2)	700 (19.8)		
1–4	1546 (48.8)	1546 (48.8)	1761 (49.9)		
≥ 5	1011 (31.9)	1011 (31.9)	1071 (30.3)		
Lifting objects over 10 kg (times)				31.088	< 0.001
0	2613 (39.0)	1148 (36.3)	1465 (41.5)		
1–5	3136 (46.8)	1502 (47.5)	1634 (46.3)		
≥ 6	948 (14.2)	515 (16.3)	433 (12.3)		
Sleep disturbance	4.64 ± 4.316	5.26 ± 4.491	4.09 ± 4.077	−11.071	< 0.001
Physical fatigue	10.80 ± 3.841	11.30 ± 3.788	10.36 ± 3.835	−9.995	< 0.001
Psychological fatigue	5.22 ± 2.546	5.40 ± 2.571	5.06 ± 2.513	−5.531	< 0.001

**Table 3:** Odds ratio (OR) and 95% confidence interval (CI) for dysmenorrhea in multivariable logistic regression (*N* = 6697).

	Model 1		Model 2		Model 3		Model 4	
	OR	95% CI	OR	95% CI	OR	95% CI	OR	95% CI
Age (years)								
20–29	1		1		1		1	
30–39	0.810⁣^∗^	0.672–0.976	0.859	0.712–1.036	0.870	0.720–1.051	0.873	0.722–1.056
≥ 40	0.374⁣^∗∗∗^	0.303–0.461	0.471⁣^∗∗∗^	0.379–0.586	0.504⁣^∗∗∗^	0.404–0.629	0.529⁣^∗∗∗^	0.423–0.662
Education								
Diploma	1		1		1		1	
Bachelor's	1.113	0.967–1.280	1.074	0.932–1.237	1.062	0.921–1.225	1.059	0.917–1.222
Master's or higher	0.997	0.837–1.187	0.971	0.814–1.160	0.987	0.826–1.180	0.989	0.826–1.183
Marital status								
Unmarried	1		1		1		1	
Married or other	0.559⁣^∗∗∗^	0.501–0.624	0.959	0.814–1.131	1.001	0.848–1.181	1.004	0.850–1.186
Annual income (USD)								
< 30,000	1		1		1		1	
30,000–39,999	1.256⁣^∗^	1.044–1.511	1.230⁣^∗^	1.021–1.483	1.190	0.987–1.436	1.142	0.944–1.382
≥ 40,000	1.142	0.960–1.359	1.133	0.950–1.351	1.075	0.901–1.283	1.000	0.831–1.203
Age of menarche (years)								
≤ 12			1		1		1	
13–14			0.890	0.784–1.011	0.910	0.800–1.035	0.915	0.804–1.042
≥ 15			0.842⁣^∗^	0.728–0.974	0.837⁣^∗^	0.722–0.970	0.842⁣^∗^	0.726–0.977
Menstrual cycle (days)								
26–31			1		1		1	
≤ 25			0.972	0.837–1.128	0.961	0.827–1.117	0.941	0.809–1.094
≥ 32			1.125	0.981–1.289	1.124	0.980–1.290	1.130	0.984–1.297
Regularity of menstruation								
Regular			1		1		1	
Irregular			0.811⁣^∗∗^	0.699–0.939	0.770⁣^∗∗^	0.663–0.894	0.752⁣^∗∗∗^	0.647–0.874
Parity								
0			1		1		1	
1			0.578⁣^∗∗∗^	0.484–0.691	0.575⁣^∗∗∗^	0.480–0.688	0.566⁣^∗∗∗^	0.472–0.678
≥ 2			0.417⁣^∗∗∗^	0.351–0.497	0.409⁣^∗∗∗^	0.343–0.487	0.408⁣^∗∗∗^	0.342–0.487
Endometriosis								
No			1		1		1	
Yes			1.737⁣^∗∗∗^	1.348–2.239	1.719⁣^∗∗∗^	1.330–2.221	1.721⁣^∗∗∗^	1.331–2.226
Uterine myomas								
No			1		1		1	
Yes			1.159	0.990–1.358	1.135	0.968–1.331	1.127	0.961–1.323
Alcohol consumption								
No					1		1	
Yes					1.121	0.998–1.259	1.130⁣^∗^	1.006–1.270
Smoking								
No					1		1	
Yes					1.115	0.814–1.527	1.111	0.810–1.190
BMI								
18.5–<23					1		1	
< 18.5					1.011	0.830–1.233	0.975	0.800–1.190
≥ 23					0.873⁣^∗^	0.782–0.975	0.877⁣^∗^	0.785–0.980
Sleep disturbance					1.056⁣^∗∗∗^	1.043–1.069	1.037⁣^∗∗∗^	1.023–1.051
Shift work								
No							1	
Yes							1.076	0.959–1.207
Weekly work hours								
≤ 40							1	
≥ 41							1.110	0.998–1.234
Standing work (hours)								
< 1							1	
1–4							0.980	0.847–1.134
≥ 5							0.922	0.779–1.091
Lifting objects over 10 kg (times)								
0							1	
1–5							1.079	0.953–1.221
≥ 6							1.230⁣^∗^	1.028–1.473
Physical fatigue							1.042⁣^∗∗∗^	1.023–1.061
Psychological fatigue							1.001	0.976–1.027
Nagelkerke *R*^2^	0.090		0.116		0.133		0.141	
Chi/df	469.774/7		140.691/9		90.065/5		45.439/8	

⁣^∗^*p* < 0.05.

⁣^∗∗^*p* < 0.01.

⁣^∗∗∗^*p* < 0.001.

## Data Availability

The datasets generated and analyzed during the current study are not publicly available. These are government data and time is required to enable data clearing and the establishment of guidelines.
